# Supplementary material for: Resolving the full spectrum of human genome variation using Linked-Reads
Source: Genome Res. 2019 Apr;29(4):635–45. doi: 10.1101/gr.234443.118 (PMC6442396; doi:10.1101/gr.234443.118)
Supplement: Supplemental Material [file supp_gr.234443.118_Supplemental_longranger-2.2.zip › longranger-2.2.tar.bz2!/lr2.2-public/lib/stan/cmdstan/stan/doc/api/html/structstan_1_1agrad_1_1fvar.html]

Stan: stan::agrad::fvar< T > Struct Template Reference


|  |  |
| --- | --- |
|  | Stan  2.5.0  probability, sampling & optimization |


- Main Page
- Related Pages
- Namespaces
- Classes
- Files

- Class List
- Class Index
- Class Hierarchy
- Class Members

All Classes Namespaces Files Functions Variables Typedefs Enumerations Enumerator Friends Macros Pages

- stan
- agrad
- fvar

Public Types |
Public Member Functions |
Public Attributes |
Friends |
List of all members

stan::agrad::fvar< T > Struct Template Reference

`#include <fvar.hpp>`

|  |  |
| --- | --- |
| Public Types | |
| typedef fvar | value\_type |
|  | |

|  |  |
| --- | --- |
| Public Member Functions | |
| T | val () |
|  | |
| T | tangent () |
|  | |
| template<typename TV , typename TD > | |
|  | fvar (const TV &val, const TD &deriv) |
|  | |
| template<typename TV > | |
|  | fvar (const TV &val) |
|  | |
|  | fvar () |
|  | |
| template<typename T2 > | |
| fvar< T > & | operator+= (const fvar< T2 > &x2) |
|  | |
| template<typename T2 > | |
| fvar< T > & | operator+= (const T2 &x2) |
|  | |
| template<typename T2 > | |
| fvar< T > & | operator-= (const fvar< T2 > &x2) |
|  | |
| template<typename T2 > | |
| fvar< T > & | operator-= (const T2 &x2) |
|  | |
| template<typename T2 > | |
| fvar< T > & | operator\*= (const fvar< T2 > &x2) |
|  | |
| template<typename T2 > | |
| fvar< T > & | operator\*= (const T2 &x2) |
|  | |
| template<typename T2 > | |
| fvar< T > & | operator/= (const fvar< T2 > &x2) |
|  | |
| template<typename T2 > | |
| fvar< T > & | operator/= (const T2 &x2) |
|  | |
| fvar< T > & | operator++ () |
|  | |
| fvar< T > | operator++ (int) |
|  | |
| fvar< T > & | operator-- () |
|  | |
| fvar< T > | operator-- (int) |
|  | |

|  |  |
| --- | --- |
| Public Attributes | |
| T | val\_ |
|  | |
| T | d\_ |
|  | |

|  |  |
| --- | --- |
| Friends | |
| std::ostream & | operator<< (std::ostream &os, const fvar< T > &v) |
|  | |

## Detailed Description

### template<typename T> struct stan::agrad::fvar< T >

Definition at line 13 of file fvar.hpp.

## Member Typedef Documentation

template<typename T>

typedef fvar stan::agrad::fvar< T >::value\_type

Definition at line 21 of file fvar.hpp.

## Constructor & Destructor Documentation

template<typename T>

template<typename TV , typename TD >

|  |  |  |  |
| --- | --- | --- | --- |
| stan::agrad::fvar< T >::fvar | ( | const TV & | *val*, |
|  |  | const TD & | *deriv* |
|  | ) |  |  |

inline

Definition at line 25 of file fvar.hpp.

template<typename T>

template<typename TV >

|  |  |  |  |  |  |
| --- | --- | --- | --- | --- | --- |
| stan::agrad::fvar< T >::fvar | ( | const TV & | *val* | ) |  |

inline

Definition at line 32 of file fvar.hpp.

template<typename T>

|  |  |  |  |  |
| --- | --- | --- | --- | --- |
| stan::agrad::fvar< T >::fvar | ( |  | ) |  |

inline

Definition at line 37 of file fvar.hpp.

## Member Function Documentation

template<typename T>

template<typename T2 >

|  |  |  |  |  |  |
| --- | --- | --- | --- | --- | --- |
| fvar<T>& stan::agrad::fvar< T >::operator\*= | ( | const fvar< T2 > & | *x2* | ) |  |

inline

Definition at line 76 of file fvar.hpp.

template<typename T>

template<typename T2 >

|  |  |  |  |  |  |
| --- | --- | --- | --- | --- | --- |
| fvar<T>& stan::agrad::fvar< T >::operator\*= | ( | const T2 & | *x2* | ) |  |

inline

Definition at line 85 of file fvar.hpp.

template<typename T>

|  |  |  |  |  |
| --- | --- | --- | --- | --- |
| fvar<T>& stan::agrad::fvar< T >::operator++ | ( |  | ) |  |

inline

Definition at line 111 of file fvar.hpp.

template<typename T>

|  |  |  |  |  |  |
| --- | --- | --- | --- | --- | --- |
| fvar<T> stan::agrad::fvar< T >::operator++ | ( | int |  | ) |  |

inline

Definition at line 118 of file fvar.hpp.

template<typename T>

template<typename T2 >

|  |  |  |  |  |  |
| --- | --- | --- | --- | --- | --- |
| fvar<T>& stan::agrad::fvar< T >::operator+= | ( | const fvar< T2 > & | *x2* | ) |  |

inline

Definition at line 42 of file fvar.hpp.

template<typename T>

template<typename T2 >

|  |  |  |  |  |  |
| --- | --- | --- | --- | --- | --- |
| fvar<T>& stan::agrad::fvar< T >::operator+= | ( | const T2 & | *x2* | ) |  |

inline

Definition at line 51 of file fvar.hpp.

template<typename T>

|  |  |  |  |  |
| --- | --- | --- | --- | --- |
| fvar<T>& stan::agrad::fvar< T >::operator-- | ( |  | ) |  |

inline

Definition at line 126 of file fvar.hpp.

template<typename T>

|  |  |  |  |  |  |
| --- | --- | --- | --- | --- | --- |
| fvar<T> stan::agrad::fvar< T >::operator-- | ( | int |  | ) |  |

inline

Definition at line 132 of file fvar.hpp.

template<typename T>

template<typename T2 >

|  |  |  |  |  |  |
| --- | --- | --- | --- | --- | --- |
| fvar<T>& stan::agrad::fvar< T >::operator-= | ( | const fvar< T2 > & | *x2* | ) |  |

inline

Definition at line 59 of file fvar.hpp.

template<typename T>

template<typename T2 >

|  |  |  |  |  |  |
| --- | --- | --- | --- | --- | --- |
| fvar<T>& stan::agrad::fvar< T >::operator-= | ( | const T2 & | *x2* | ) |  |

inline

Definition at line 68 of file fvar.hpp.

template<typename T>

template<typename T2 >

|  |  |  |  |  |  |
| --- | --- | --- | --- | --- | --- |
| fvar<T>& stan::agrad::fvar< T >::operator/= | ( | const fvar< T2 > & | *x2* | ) |  |

inline

Definition at line 95 of file fvar.hpp.

template<typename T>

template<typename T2 >

|  |  |  |  |  |  |
| --- | --- | --- | --- | --- | --- |
| fvar<T>& stan::agrad::fvar< T >::operator/= | ( | const T2 & | *x2* | ) |  |

inline

Definition at line 104 of file fvar.hpp.

template<typename T>

|  |  |  |  |  |
| --- | --- | --- | --- | --- |
| T stan::agrad::fvar< T >::tangent | ( |  | ) |  |

inline

Definition at line 19 of file fvar.hpp.

template<typename T>

|  |  |  |  |  |
| --- | --- | --- | --- | --- |
| T stan::agrad::fvar< T >::val | ( |  | ) |  |

inline

Definition at line 18 of file fvar.hpp.

## Friends And Related Function Documentation

template<typename T>

|  |  |  |  |
| --- | --- | --- | --- |
| std::ostream& operator<< | ( | std::ostream & | *os*, |
|  |  | const fvar< T > & | *v* |
|  | ) |  |  |

friend

Definition at line 140 of file fvar.hpp.

## Member Data Documentation

template<typename T>

T stan::agrad::fvar< T >::d\_

Definition at line 16 of file fvar.hpp.

template<typename T>

T stan::agrad::fvar< T >::val\_

Definition at line 15 of file fvar.hpp.

---

The documentation for this struct was generated from the following file:

- src/stan/agrad/fwd/fvar.hpp


---

[ Stan Home Page ]

*© 2011–2014,
Stan Development Team.*
